# Evidence for protective and deleterious microglial phenotypes in an individual underscores microglia as an attractive therapeutic target

**DOI:** 10.1002/alz70856_103454

**Published:** 2025-12-26

**Authors:** Paul Edison

**Affiliations:** ^1^ Division of Neurology, Department of Brain Sciences, Imperial College London, United Kingdom, London, London, United Kingdom

## Abstract

**Background:**

Microglia can take on proinflammatory or anti‐inflammatory phenotypes, but it is unclear how these phenotypes play out along the Alzheimer's disease (AD) continuum. The purpose was to assess regional variances of microglial activation in distinct stages of the disease, and the role of microglial responses on grey matter volume and mean diffusivity in different brain areas and on cognition across the course of AD.

**Method:**

48 subjects (23 AD patients, 14 mild cognitive impairment [MCI], and 11 healthy controls [HC]) underwent TSPO‐PET, diffusion tensor imaging (DTI) and extensive neuropsychometric assessment. SPM (Statistical parametric mapping) analysis was conducted for single subject analysis. GM volume for each subject was derived from T1 volumetric MRI using the FreeSurfer pipeline, mean diffusivity (DTI), alongside voxel‐based morphometric analysis. Using postmortem brain tissue from 26 AD subjects, we evaluated CD32a and CD163 anti‐bodies, markers of proinflammatory and anti‐inflammatory microglia, respectively.

**Result:**

Voxel‐wise analyses identified positive and negative clusters of associations for IRF90 with GM volume and mean diffusivity. The presence of pro‐ and anti‐inflammatory phenotypes was confirmed in human postmortem brain, in the frontal, parietal and entorhinal cortices. In addition, higher TSPO, signal in temporal lobes associated with lower hippocampal volume and mean diffusivity, and higher TSPO, signal across the cortex, correlated with poorer performance on neuropsychometric tests.

**Conclusion:**

There may be protective and deleterious microglial phenotypes in an individual, region specific and disease stage dependent. Therapeutic modulation of microglia is warranted to promote anti‐inflammatory functions while suppressing pro‐inflammatory functions.

